# Nitrous Oxide, or “Laughing Gas,” as an Anæsthetic

**Published:** 1864-02

**Authors:** E. Andrews

**Affiliations:** Professor of Surgery in Chicago Medical College


ARTICLE VIII.

### NITROUS OXIDE, OR "LAUGHING GAS," AS AN ANÆSTHETIC.

BY E. ANDREWS, M.D., Professor of Surgery in Chicago Medical College.

Considerable stir has been made among the dentists of late by the introduction of a new anæsthetic, which is nothing else than the old-fashioned laughing gas of the chemists. As is usual in such matters, the quacks of tooth-pulling, make the most noise about it, while the respectable men use it with much less enthusiasm. Perhaps the most amusing thing about it is that a large portion of the second-rate dentists have been victimized by the introducers of the gas, and induced to pay fifty dollars apiece for the "*secret*," and for a small package of Nitrate of Ammonia, with which to commence operations. In some handbills distributed around the city it is called by the sounding title of Muhrite of Oxygen, and is extolled as being entirely free from the dangers of chloroform. The gas thus heralded, is simply the old-fashioned protoxide of nitrogen, otherwise called nitrous oxide, and laughing gas. The term "Muhrite of Oxygen," is a sheer fabrication, there being no such name or compound in chemistry. The article is prepared by heating nitrate of ammonia in a glass retort. The salt is decomposed by the heat, and converted into nitrous oxide and water. It is passed through a water bath to free it from any impurities that may come over with it, and then collected in a large gasometer. For the purpose of inhalation, it is drawn off into india rubber gas bags, holding three to five gallons each. Although, as above mentioned, the dental quacks make the most noise about it, still the article is more or less used by all the principal dentists of the city. As everything which possesses claims as an anæsthetic is of importance to the surgeon, I thought it my duty to examine into its qualities, and am indebted to Drs. J. C. FULLER, J. C. DEAN, and W. W. ALLPORT, dentists in this city, for special facilities in pursuing my inquiries. To begin with, I took a moderate portion of the gas myself, in order to judge of the sensations produced by it. The first effect was a sense of buzzing and vibration over the whole system, precisely like that produced by chloroform, which increased rapidly to such an uproarous degree, that the senses of hearing, feeling, and seeing, seemed to be rapidly becoming lost and overwhelmed in a tremendous roaring, jaring, and dizziness. At this point I was aware that I was drawing in and throwing out my breath in a furious manner, without any reason, and without conscious effort. By pinching the flesh of my hand I perceived that I had still, to some degree, the power of feeling pain, though all the senses were blunted. As I did not wish to proceed to entire unconsciousness, I then withdrew the tube from my mouth, having been inhaling about two minutes. The effect subsided in about two minutes more, the return to full consciousness being accompanied by a free perspiration and a mild exilaration of the feelings. The pulse at the beginning was 84 per minute, and in the height of the effect about one hundred. I then took a larger quantity and administered it to a patient to pretty full anæsthesia. The pulse at the beginning of the experiment was 85 per minute. In abou. a minute it had risen to 100, with diminished force, and a free perspiration broke out on the forehead. The patient now began to look blue in the lips, showing obvious marks of incipient asphyxia. At the same time he began to make his inspirations and expirations in a furious and noisy manner. The pulse by another minute had increased to 120 per minute, and was feebler. The perspiration was more copious, and the blue asphyxiated appearance of the countenance was so decided that I judged it prudent to withdraw the gas, and terminate the experiment. The anæsthesia was perfect, the patient not shrinking from the severest pinches, and not remembering any pain from them afterwards. The anæsthesia continued about 45 seconds, when the patient began to laugh violently, and finally came to his consciousness with an exulting yell, and a loud smack of his fists. Ordinarily the patient makes but little trouble by any boisterousness, and seldom feels any depression, nausea, or other trouble afterwards.

A man named COLTON, an itinerant lecturer on Chemistry, who has traversed the country for many years in that capacity, claims the credit of having demonstrated the anæsthetic power of this agent to the world, while he assigns the original discovery of its effects to a Dr. WELLS. COLTON claims to have given it to patients for the extraction of over eight thousand teeth, and without injury in any instance. There are, however, cases on record of headache and delirium following its use, the delirium in a few cases lasting months, and it is asserted that deaths have resulted from it, but I have not been able to obtain decisive authority for the statement, except in one instance. Animals, however, have been killed by it. So far as we can judge at present, the use of nitrous oxide would seem, like all other anæsthetics, to be accompanied by a slight but positive danger. The blueness of the lips indicating incipient asphyxia, is present in every instance, and it is not probable that such a state can be induced upon all sorts of patients, and in all pathological conditions with entire safety, still I know of no reason at present for supposing that it is any more dangerous than chloroform, and it is certainly much more agreeable to take.

For the purposes of general surgery, the nitrous oxide will not be available. The chief difficulty is the extremely transitory nature of its effects. Having only from 80 to 90 seconds of insensibility in which to operate, the surgeon can seldom use the gas, for if he should continue the inhalation without interruption for many minutes, death would probably ensue from asphyxia, as the nitrous oxide seems to have no power to arterialize the blood. Still in operations which require only a moment for their performance, such as lancing felons and abscesses, it might be easily used. In some instances, also, it could be given repeatedly at one sitting, the patient being allowed to recover from one inhalation before proceeding to another, and the operation completed by successive stages.

It has both conveniences and inconveniences connected with it. Of the former, is the fact that scarcely any time is required to produce its full effects, which is an important item with the surgeon, and also that there is no nausea and vomiting afterwards, which is a comfort to the patient. On the other hand, it is a difficult thing to preserve and carry, and the practitioner going to see a patient with it, must take with him a five gallon gas bag full, which he can by no means put into a corner of his pocket.

It is probable, therefore, that nitrous oxide will be permanently established as a dental anæsthetic, but will only be occasionally used in general surgery.

